# Spanish Adaptation of Meaning-Centered Psychotherapy for Participants With Cancer: Study Protocol of a Randomized Control Trial

**DOI:** 10.3389/fpsyt.2022.892573

**Published:** 2022-07-07

**Authors:** Jose Heliodoro Marco, Pilar Llombart, Verónica Guillén, Rosa M. Baños, Rocio Romero, Ana Garcia-Conde, Sandra Pérez Rodríguez

**Affiliations:** ^1^Department of Personality, Assessment, and Psychological Treatments, University of Valencia, Valencia, Spain; ^2^CIBER Fisiopatología Obesidad y Nutrición (CIBEROBN), Madrid, Spain; ^3^School of Doctorate, Catholic University of Valencia San Vicente Mártir, Valencia, Spain; ^4^Department of Psychology, Instituto Valenciano de Oncologia, Valencia, Spain; ^5^Department of Personality, Assessment, and Therapeutic Interventions, Catholic University of Valencia San Vicente Mártir, Valencia, Spain

**Keywords:** Meaning-Centered Psychotherapy, cancer, meaning in life, cognitive behavioral therapy, logotherapy

## Abstract

**Background:**

Meaning-Centered Psychotherapy (MCP) is effective in improving meaning in life, hope, optimism, self-efficacy, well-being, and quality of life, and in reducing stress in people with cancer. However, all the studies on the application of MCP in cancer patients have been carried out in Anglo-Saxon samples. Therefore, it is necessary to adapt and verify the efficacy of MCP in populations that speak languages other than English, such as Spanish. Moreover, to expand the data supporting the efficacy of MCP for cancer patients, it would be necessary to compare MCP to other active therapies such as Cognitive Behavioral Therapy (CBT).

**Methods:**

The aims of the proposed study are: the first objective is to verify the efficacy of the MCP intervention for Spanish participants with cancer in a randomized control trial (RCT) comparing it to CBT. The second objective is to analyze the feasibility and acceptance of MCP in Spanish participants with cancer. The third objective is to analyze whether the changes produced in the meaning in life dimensions (presence, search, comprehension, purpose, and mattering) will predict changes in anxiety, depression, quality of life, etc. Our research team adapted MCP for Spanish participants with cancer. This paper presents the study protocol. The study design consists of a two-arm RCT with two conditions: MCP and CBT, where participants will be randomized to one of the two groups. Eligible participants will be adults with stage I, II, and III cancer who were treated with curative intent and had completed their main medical treatment (surgery, radiotherapy, or chemotherapy). Participants will be assessed at pretreatment, post-treatment, and 6-month follow-up. The intention-to-treat principle will be used when analyzing data, using mixed-effects models with full information and maximum likelihood estimation.

**Discussion:**

This study will provide results that confirm the efficacy of the MCP in Spanish participants with cancer.

**Trial Registration:**

ClinicalTrials.gov; https://register.clinicaltrials.gov/prs/app/template/Home.vm?uid=U0005WS9&ts=4&sid=S000BOTT&cx=bvr2ue, identifier NCT05197348

## Introduction

Being diagnosed with and coping with cancer is one of the most difficult and challenging life experiences a person can have. People with cancer have twice as much psychopathological distress as the cancer-free population, and the levels of depression in survivors in the first year after treatment are very high compared to the general population (1). In addition, anxiety, fatigue, sadness, emotional, and physical pain, poor quality of life, hopelessness, desire for hastened death, and low meaning in life are common negative emotional states in people with cancer and survivors (2–4).

Studies in clinical and non-clinical samples of patients without a cancer diagnosis have shown that meaning in life is an emotional state that is negatively associated with hopelessness, anxiety, emotional dysregulation, coping capacity, and depression, and it is a buffer against psychopathology (5, 6). Specifically in people with cancer, previous studies have found that meaning in life could buffer against depression, hopelessness, and the desire for hastened death (7). Therefore, previous studies suggest that intervening in meaning in life in cancer patients can be an effective treatment to improve the aforementioned negative emotional states and lead to better coping with life after a cancer diagnosis (8, 9).

Meaning-Centered Psychotherapy (MCP) is a logotherapeutic-oriented psychotherapy based on Viktor Frankl's (10, 11) principles and premises, such as the will to meaning, meaning in life, responsibility, freedom, finding meaning through courage and commitment, living one's legacy, and finding a sense of meaning through the three sources of meaning: creative, experiential, and attitudinal sources. MCP has been utilized in several samples and settings, and a meta-analytic study found that MCP is effective in improving meaning in life, hope, optimism, self-efficacy, wellbeing, and quality of life, and in reducing stress (12).

The MCP application for cancer patients was initially developed by Breibart as an eight-session group therapy for patients with advanced stage III-IV cancer (8). In a randomized control trial (RCT), it was shown to be more effective than supportive psychotherapy in improving spiritual wellbeing, sense of meaning, and anxiety, and the treatment effects of MCP were stronger 2 months after treatment. However, there were no significant differences in the participants' depression. Subsequently, Breibart (13), in an RCT with patients with stage III or IV cancer, adapted the MCP to seven sessions to be applied individually. Individual MCP was compared with a control group (massage therapists), and the results showed that MCP produced a greater improvement in meaning in life, spiritual wellbeing, faith, physical symptoms of distress, burnout, and quality of life. However, the improvement was not sustained at 2 months. The same authors indicated that there were no statistically significant improvements in depression or anxiety. The authors suggested that MCP may be more effective in a group format than in an individual format. MCP was later adapted by Rosenfeld (14) for palliative care (MCP-Palliative Care) by reorganizing the contents of the original MCP to reduce it to only three sessions. A pilot study was conducted on the feasibility, acceptability, and perceived utility of MCP in 12 patients with life expectancies of <6 months, and satisfactory opinions were obtained about the acceptability and understanding of MCP.

MCP was also applied to cancer survivors (9) in an RCT with a 6-month follow-up, where the MCP condition was compared with two other conditions: (a) a Supportive Group and (b) Treatment as Usual (TAU) with their general practitioner. The results at the 6-month follow-up showed that the MCP treatment improved personal meaning, positive relations, purpose in life, a fighting spirit, hopelessness, goal-orientedness, and psychological wellbeing and adjustment, and it reduced psychological distress and depressive symptoms more than the TAU condition. MCP was more effective in improving goal-orientedness and a fighting spirit compared to the Supportive Group, as well as personal growth and environmental mastery at the 6-month follow-up. In addition, MCP has also been used with people with grief (15) as the Meaning-Centered Grief Psychotherapy (MCGP) for parents who lost a child to cancer. MCGP is an individual and manualized intervention consisting of 16 sessions for people with prolonged grief. In this pilot study, after the intervention, patients showed improvements in grief, depression, meaning in life, positive affect, post-traumatic growth, and quality of life. Finally, MCP has been adapted for caregivers of people with cancer (16), adolescent and young participants with cancer (17), and participants with advanced cancer (4, 18).

As we have seen, MCP has been shown to be effective in improving meaning in life and quality of life and reducing distress in people with cancer. However, it is important to emphasize that all the studies mentioned above on the application of MCP in cancer patients have been carried out in Anglo-Saxon samples and have mainly focused on patients with a diagnosis of cancer in III-IV stages. The construction of meaning, vital goals, responsibility, sources of meaning, and the comprehension and acceptance of these constructs depend on cultural, social, and religious factors, etc. (19). There are studies that indicate that the construction of meaning and growth in the face of adverse situations is different for Anglo-Saxon and Spanish populations. For example, a previous study found that, in the American sample, the presence and search for meaning were moderately and negatively associated, whereas these two factors were uncorrelated in the Spanish population (20). Therefore, it is necessary to adapt and verify the efficacy of MCP in populations that speak languages other than English, such as Spanish and to test the efficacy of MCP in patients with an early stage I–III diagnosis of cancer.

Another psychotherapy that has been found to be effective in participants with cancer is cognitive behavioral therapy (CBT). The adaptation of CBT to participants with cancer is a comprehensive cognitive-behavioral model based on Beck's therapy for depression. The cognitive model of adjustment to cancer states that the patient's assessment, interpretation, and evaluation of cancer is what will determine his/her emotional and behavioral reaction. CBT has been considered a psychological behavioral therapy that, when specifically delivered to cancer patients, focuses on managing emotions, cognitions, problem-solving, relaxation, and assertiveness, and on improving coping with cancer (21). This therapy has been found to be effective in improving anxiety, depression, helplessness, fatalism, anxious preoccupation, and coping (22). CBT has also been found to improve the change in quality-of-life associated with distress due to medical treatments or to adjustment after the disease is treated (23) and lead to greater adaptability to cancer, thus reducing vital anguish (24).

As we can see in the aforementioned RCTs, MCP has been compared to waiting list, TAU with the general practitioner, and active treatments such as supportive therapy. To expand the data supporting the efficacy of MCP for cancer patients, it would be necessary to compare MCP to other active therapies such as CBT. According to the data, no previous studies have compared MCP to active treatments such as CBT for cancer patients, which is the general objective of this study.

The aims of the proposed study are: the first objective is to verify the efficacy of the MCP intervention for Spanish participants with cancer in an RCT comparing it to CBT. The second objective is to analyze the feasibility and acceptance of MCP in Spanish participants with cancer. The third objective is to analyze whether the changes produced in the MIL dimensions (presence, search, comprehension, purpose, and mattering) will predict changes in anxiety, depression, quality of life, etc.

We propose the following hypothesis: (a) After the intervention, all the participants in the two treatment conditions (MCP vs. CBT) will improve on MIL, anxiety, depression, and quality of life; however, the participants in the MCP condition will improve more than those in the CBT condition; (b) After the intervention, both interventions will have good acceptance by the participants, but the MCP condition will have greater acceptance than the CBT condition; (c) The changes produced in the MIL dimensions (presence, search, comprehension, purpose, and mattering) after the intervention will be related to changes in anxiety, depression, and quality of life.

In this article, we present the study protocol.

## Methods and Design

### Study Design

**Figure 1:**
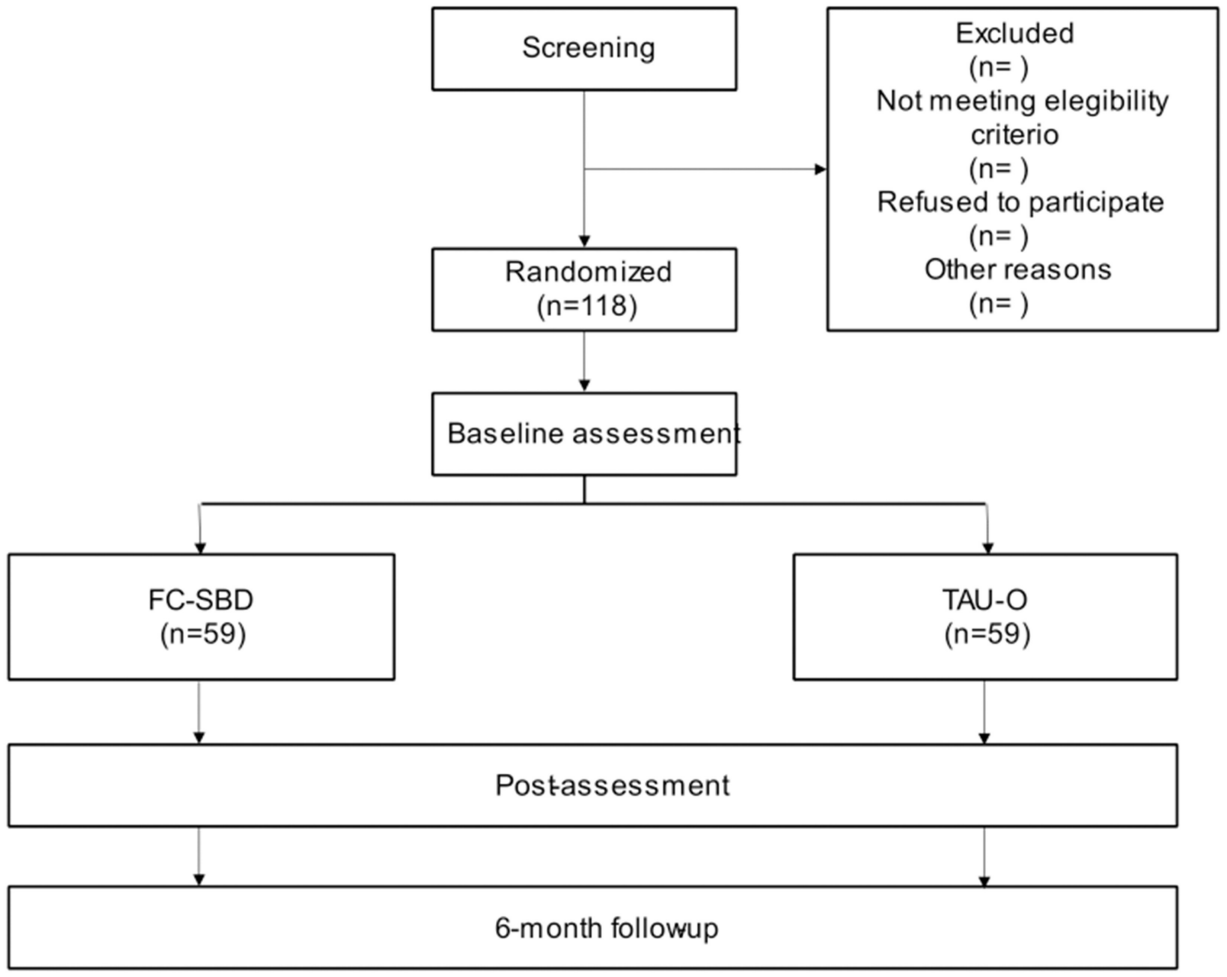
Flowchart of the study.

The study design consists of a two-arm RCT with two conditions: MCP and CBT, where participants will be randomized to one of the two groups. Figure 1 contains the flow chart. For this protocol, we will follow the CONSORT statement (Consolidated Standards of Reporting Trials, http://www.consort-statement.org) (25, 26) and the SPIRIT guidelines (Standard Protocol Items: Recommendations for Interventional Trials (27, 28).

### Sample Size

The sample size for this study was generated from effect sizes found in other studies on MCP in participants with cancer, mainly meta-analyses. Dietrich (29) found medium effect sizes for meaning in life (*d* = 0.81), Quality of life (*d* = 0.60), anxiety (*d* = −0.47), and depressive symptoms (*d* = −0.50). These effect sizes are consistent with other studies on MCP in psychological treatments for other mental disorders, such as a meta-analysis by Vos et al. (12) on existential psychotherapies. Based on the results of these studies, we expect an effect size of 0.60 because the study design consists of two experimental groups. To calculate the sample size, we used the G^*^Power 3.1 software (30), taking into account an alpha of 0.05 and a statistical power of 0.80 on a two-tailed *t*-test. We need a sample size of 90 participants (45 participants per condition) to reach an effect size of 0.60 for meaning in life. Finally, we have considered possible sample loss during the treatment program, and based on studies of interventions with MCP, we expect a 30% dropout rate (9). Therefore, taking this outcome into account, our study requires a total sample size of 118 participants (59 participants per experimental condition).

### Participants

Eligible participants will be adults with stage I, II, and III cancer who were treated with curative intent and had completed their main medical treatment (surgery, radiotherapy, or chemotherapy). Moreover, participants will have to express a need for psychological care, and they will have low or undefined meaning in life (scores below 105 on the Purpose in Life test) (31). The recruitment will be carried out by an oncologist and two clinical psychologists with more than 10 years of experience in cancer care. The sample will be recruited from the Valencian Oncology Institute in Valencia from January to June 2022. Thus, it is an ongoing study.

The exclusion criteria will be: (1) participants who are currently receiving another psychological or psychiatric treatment; (2) the diagnosis of a serious mental disorder (schizophrenia, substance dependence, dementia, or cognitive impairment).

### Procedure

Cancer survivors who have been treated at the Valencian Institute of Oncology will be offered the opportunity to participate in the study. Once they have signed the informed consent, a clinical psychologist who is an expert in cancer will carry out two evaluation sessions to verify that they meet all the inclusion criteria. Once evaluated, randomization will be carried out by an independent researcher who is blind to the evaluation of the participants. At all times, the randomization sequence will be concealed from the clinicians participating in the study. The evaluation will be carried out by independent evaluators who are blind to the experimental conditions. Subsequently, each participant will begin their group intervention in the condition to which they have been assigned. Participants will not receive any other psychological or psychiatric treatment during this study. When the psychotherapy is over (MCP or CBT), the evaluation will be carried out again. A follow-up will be carried out at 1 month, at 3 months, and at 6 months. The evaluation will be carried out online before and after finishing the treatment and in the follow-ups. All the interventions will be performed by clinical psychologists or general health psychologists with a Master's degree and previous training in the application of the programs. All the interventions will be carried out through online video conferences in both treatment conditions.

### Ethics

The study involves clinical experimentation with human participants. Researchers adhere to the Helsinki Convention and the Madrid Declaration of the World Psychiatric Association on clinical research. This project has been approved by the Ethics and Research Committee of the Valencian Institute of Oncology Foundation (CEIM: 2019–17). The study was registered with Clinicaltrials.gov as NCT05197348. All participants will be volunteers and give their informed consent to participate in the study. All eligible participants will receive oral and written information about the study and the two intervention modalities. Specifically, they will be informed that they can leave the study at any time without any explanation, and that this decision will not affect the continuation of their usual treatment at the Valencian Institute of Oncology. The selection and evaluation of the participants will be carried out by a clinical psychologist who is blind to the condition assigned to the participants. The intervention will be carried out by two psychologists who specialize in cancer. In addition, it should be noted that the protocols for action and custody of the information followed in the Valencian Institute of Oncology, where the participants will be recruited and the interventions will be carried out, comply with all the requirements of the LOPD (Organic Law 15/1999, of December 13, 2018). In addition, based on the existing knowledge in this field, we have not found any risks to the participants. However, any important clinical change that involves some type of risk will not only imply the participant's termination in the study, but also his/her referral for study and specialized care. The evaluation protocol described above is composed of standardized and risk-free instruments for the participants (interviews and questionnaires). The intervention protocols are based on empirically validated treatments designed and developed by personnel with extensive knowledge and experience in this field. For ethical reasons, participants in the CBT condition will be offered the opportunity to receive the MCP program, if they so desire, even if the study has already ended.

### Interventions

#### Meaning-Centered Group Psychotherapy

To carry out the adaptation of the Manualized Meaning-Centered Group Psychotherapy (MCP) group format for patients with advanced cancer (32) to Spanish, we used the ecological validity model (EVM) (33) as a framework for the cultural adaptation of MCP to respond to the needs of the Spanish population with cancer. According to the EVM, to adapt an intervention for a new cultural group, seven dimensions need to be addressed: language, context, persons, metaphors, concepts, goals, and methods. We first carried out a translation of the original MCP program from English to Spanish. The translation was performed by two bilingual clinical psychologists who are experts in meaning-focused psychotherapy; this translation was supervised by a bilingual editor with expertise in clinical psychology. A feasibility study (in preparation) with 12 participating cancer survivors was conducted by two clinical psychologists who are experts in cancer. Finally, we tested the understanding, acceptance, and feasibility of the Spanish version of the MCP with a qualitative interview and questionnaires. The intervention lasts 2 months and includes 8 sessions that follow a 2-h group format on a weekly basis. We will follow the manualized MCP for patients with advanced cancer (32). The MCP program is divided into 8 sessions:

Session 1: Concepts and Sources of Meaning.
Introductions; review of concepts and sources of meaning; Meaningful Moments experiential exercise; homework is to read Man's Search for Meaning and reflect on the Session 2 experiential exercise.
Session 2: Cancer and Meaning.
Discussion of sense of identity before and after cancer diagnosis; Who am I? experiential exercise; homework is to reflect on the Session 3 experiential exercise.
Session 3: Historical Sources of Meaning (Past Legacy).
Discussion of life as a legacy that has been given (past); Historical Sources of Meaning-Past experiential exercise; homework is to reflect on the Session 4 experiential exercise.
Session 4: Historical Sources of Meaning (present and future).
Creation of lifeline. Own legacy development. Exercises: Reflection on the autobiography (in session and at home). Evaluate how situations are approached, objectives they had and that have been achieved. Explore a future legacy.
Session 5: Attitudinal Sources of Meaning: Encountering Life's Limitations
Discussion of confronting limitations imposed by cancer, prognosis, and death; Encountering Life's Limitations experiential exercise; introduction to Legacy Project; homework is to reflect on the Session 6 experiential exercise.
Session 6: Creative Sources of Meaning: Engaging in Life Fully.
Discussion of creativity, courage, and responsibility; Creative Sources of Meaning experiential exercise; homework is to reflect on the Session 7 experiential exercise.
Session 7: Experiential Sources of Meaning: Connecting with Life
Discussion of experiences as sources of meaning, such as love, nature, art, and humor; Love, Beauty, and Humor experiential exercise; homework is to complete the Legacy Project for presentation in Session 8.
Session 8: Transitions: Reflections, and Hopes for the Future.
Review of sources of meaning as resources, reflections on lessons learned; Hopes for the Future experiential exercise; goodbyes.

#### Cognitive Behavioral Psychotherapy

The intervention lasts 2 months and includes 8 sessions that follow a 2-h group format on a weekly basis, with the following sessions (21):

Session 1: Presentation of psychotherapy, establishing the goals of psychotherapy. Presentation of the participants. Updated information about psychological consequences of cancer.
Session 2: Increase in enjoyable activities. Behavioral activation. Progressive muscle relaxation training. Slow breathing training.
Session 3: Cognitive model of coping with cancer. ABC model. Psychoeducation on negative thoughts. Training in detecting negative thoughts. Presentation of cognitive distortions.
Session 4: Training in cognitive restructuring techniques.
Session 5: Training in problem-solving skills.
Session 6: Being aware of our own needs. Self-care. Assertiveness skills training.
Session 7: Setting goals for the future.
Session 8: Summary, relapse prevention, and end of psychotherapy.

### Measures

After reviewing the literature, an evaluation protocol was designed that includes the main instruments that have been commonly used by authors working in this field (29), as well as some additional instruments that we consider relevant to the hypotheses proposed. In all cases, the Spanish validations of the assessment instruments will be used; if they do not exist, the validations will be carried out by the researchers of this study. Participants in both treatment conditions will complete all primary and secondary outcome measures at five time points: before and after treatment and at 1-month, 3-month and 6-month follow-ups.

#### Measures

Data on demographic variables: Age, gender, educational level, marital status, cancer diagnoses, cancer treatment, mental diagnoses.

##### Measures of Primary Outcomes

*Meaning in Life Questionnaire [MLQ* *(**34**)]*. The MLQ is a self-reported questionnaire made up of 10 items, and it was developed to assess the two main dimensions of meaning in life: presence and search for meaning in life. The items are rated on a 7-point scale ranging from 1 (absolutely false) to 7 (absolutely true). The factors of Presence and Search were correlated (*r* = −0.19), and internal consistency was good for Presence (0.86) and Search (0.87). One-month test-retest reliability coefficients were 0.70 for Presence and 0.73 for Search.

*The Multidimensional Existential Meaning Scale [MEMS* *(**35**)]*. The MEMS assesses the MiL dimensions: Comprehension, purpose, and mattering, with a total of 15 items (e.g., “My life makes sense”; “I have overarching goals that guide me in my life”; “I understand my life”; “I know what my life is”). Likert type responses are given on a 7-point scale (1 = Very strongly disagree; 7 = Very strongly agree). The three MEMS subscales showed adequate internal consistency: Comprehension (ϖ = 91), Purpose (ϖ = 92), and Mattering (ϖ = 86).

##### Measures of Secondary Outcomes

*Overall Anxiety Severity and Impairment Scale [OASIS* *(**36*, *37**)]*. The OASIS is a five-item instrument that assesses the frequency and intensity of anxiety symptoms during the past week. In addition, it measures interference in work and academic, social, and daily life domains, as well as avoidance behaviors. The items are rated on a Likert-type scale (0–4). The psychometric properties are good in terms of internal consistency (α = 0.86), convergent and discriminant validity, and sensitivity to change (α = 0.86).

*Overall Depression Severity and Impairment Scale [ODSIS* *(**38*, *39**)]*. The ODSIS is a six-item questionnaire that assesses the frequency and intensity of depression symptoms during the past week. In addition, it measures interference in work and academic, social, and daily life domains, as well as avoidance behaviors. The items are rated on a Likert-type scale (0–4). The psychometric properties are good in terms of internal consistency (α = 0.93).

*Hopelessness Scale [HS* *(**40**)]*. It is a questionnaire that measures the level of hopelessness. It is made up of 20 items with dichotomous responses (True or False). It presents adequate internal consistency (α = 0.93) and has been validated in the Spanish population (41).

*The Positive and Negative Affect Schedule [PANAS* *(**42*, *43**)]*. The questionnaire includes 20 adjective items, 10 assessing positive affect (PANAS-P) and 10 assessing negative affect (PANAS-N). Respondents are asked to rate the extent to which they experienced each emotion within a specified time period, using a 5-point scale (1 = Very slightly or not at all; 5 = Very much). Both affect subscales showed adequate internal consistency: PANAS-P (α =0.89) and PANAS-N (α =0.91).

*Quality of Life Index-Spanish Version [QLI* *(**44*, *45**)]*. It is a 10-item index of perceived quality of life. It refers to physical and emotional wellbeing, functioning at work, personal relationships, self-independence, support in the community and from an emotional point of view, spiritual wellbeing, and overall perceived quality of life. The items are rated on a Likert scale (0–10) where higher scores indicate higher perceived quality of life. The psychometric properties are good for both internal consistency (α =0.89) and test-retest reliability (*r* = 0.87).

*Opinion and Expectations of Treatment Scale [OTSM* *(**46**)]*. This scale was designed and developed by members of the research team, based on an adaptation of another opinion and expectations questionnaire (46). The constructs this scale assesses are: opinion, acceptance and satisfaction with the skills training program, and changes in the participants after completing each module. The questions refer to the rationale for the intervention, recommendations for the program, satisfaction with the program, and usefulness and expectations of the skills training. The items are rated on a Likert-type scale ranging from 0 “Not at all” to 10 “Very much.”

*Mini-Mental Adjustment to Cancer Scale [MINI-MAC* *(**47**)]*. The MINI MAC evaluates the different ways of coping in cancer patients. It is a self-rating questionnaire made up of 29 items that measure five dimensions of coping: fighting spirit, helplessness-hopelessness, anxious preoccupation, fatalism, and cognitive avoidance. The items are rated on a Likert scale (1–4). The Spanish version of the MINIMAC (48) showed good psychometric properties for all the subscales (fighting spirit α =0.60, helplessness-hopelessness α =0.82, anxious preoccupation α =0.90, fatalism α =0.70, and cognitive avoidance α =0.80).

*The Posttraumatic Growth Inventory [PTGI* *(**49*, *50**)]*. The PTGI is a 21-item questionnaire that assesses the perception of personal benefits in survivors of a traumatic event. A Likert response format with six categories is used, with scores ranging from 0 (no change) to 5 (very high degree of change) in a positive sense; the higher the score, the greater the change perceived. The PTGI is composed of five dimensions: relating to others, new possibilities, personal strength, spiritual change and a better understanding of spiritual matters and stronger religious beliefs, and a new appreciation of life. The PTGI shows good internal consistency (α =0.80).

### Data Analyses

For data analysis, the Consort guidelines will be followed. Prior to analyzing whether MCP was more effective than CBT, we will verify that the two experimental conditions are similar in all the sociodemographic variables, such as age, sex, marital status, and education. In addition, we will verify that the two conditions are similar in terms of the percentage of participants who have received chemotherapy, radiotherapy, and surgery. To do so, we will use comparison of means (Student's t) for the continuous variables and chi square for the categorical variables. Moreover, the intention-to-treat principle will be used when analyzing pre- and post-treatment data and at the 6-month follow-up, using mixed-effects models with full information and maximum likelihood estimation. This method has been recommended due to its flexibility in handling missing data (51). To complement the MANOVA results and *post hoc* comparisons, effect sizes will be calculated using the standardized mean difference proposed by Cohen (52). These effect sizes will be calculated to assess changes within and between groups, all based on a pooled standard deviation. MANCOVAS will be carried out to find out if there are differences in the participants' response to the two experimental conditions depending on the type of medical treatment administered (surgery, chemotherapy, or radiotherapy), the stage of the cancer before the start of medical treatment, and whether the patient has a relapse during the psychological treatment. Moderation and mediation analyses will be performed to investigate possible mediators of change during treatment, such as meaning in life or coping style. Although per-protocol analyses (only analyses of data from participants who complete the treatment) suffer from selection bias, they will also be conducted because they allow conclusions to be drawn about the maximum efficacy of the intervention in participants who are fully compliant with treatment. However, when the trial is completed, the analytical methodology for controlled clinical trials will be reviewed before analyzing the data to select the most appropriate analytical procedures.

For the qualitative study, semi-structured in-depth interviews will be used. The research design will be carried out following the criteria established by Cooke, Smith, and Booth (53). Data will be analyzed using the consensual qualitative research method. Data reporting will be carried out following the COREQ guidelines (54).

## Discussion

The first objective of this proposed study will be to analyze the efficacy of the Spanish adaptation of MCP for cancer survivors. For this aim, we have designed an RCT in which we will compare MCP to CBT in people with cancer, with three assessment times: pre-treatment, post-treatment, and 6-month follow-up. In previous studies on the efficacy of MCP, it has been compared to waitlist, TAU, therapeutic massage, and supportive social therapy (8, 9, 12, 13). In order to broaden the knowledge about the efficacy of MCP, in this study we compare it to CBT. In previous studies, CBT has shown its effectiveness in reducing anxiety and depression, facilitating cancer adjustment, and improving quality of life (21, 22). Therefore, if MCP is equally effective or more effective in reducing anxiety and depression and improving quality of life, hopelessness, and meaning in life, this result would suggest that MCP could be a treatment of choice for people with cancer.

The second objective of this proposed study will be to analyze the feasibility and acceptance of MCP in Spanish participants with cancer. The concepts and premises of logotherapy, such as meaning-making, vital goals, the concepts of responsibility, freedom, courage, legacy, and will to meaning, or meaning in life, are subject to cultural, religious, and social factors specific to each culture (19). Given that all the RCTs on the efficacy of MCP have been carried out in an English-speaking population, it is necessary to analyze the extent to which the Spanish participants accept the program and are comfortable with it, as well as their opinions and expectations about it, in order to improve adherence and future implementation.

The third objective of this proposed study will be to analyze whether the changes produced in the MIL dimensions (presence, search, comprehension, purpose, and mattering) predict the changes in anxiety, depression, quality of life, etc. In this study, we will use multidimensional measures of meaning to assess change in meaning in life. The MEMS (35) will allow us to evaluate the three dimensions of meaning (comprehension, purpose, and mattering), and the MLQ (36) will allow us to include two other dimensions (presence, search). Evaluating the effectiveness of MCP with multidimensional measures will allow us to find out whether it is effective for all the dimensions of meaning or, on the contrary, produces a change in one dimension (e.g., presence) but not in another (e.g., search). In addition, we will be able to verify which dimension of meaning best predicts the change in the patients' clinical measurements.

Another contribution of this proposed study is the application of MCP through videoconference. To the best of our knowledge, there are no studies on the efficacy of PCM *via* videoconference. This would allow us to take MCP to people who live in places where there are no psychotherapists, so that a greater number of people from very distant places could receive treatment. Some studies have described the advantages of online psychosocial interventions (55), such as compensating for geographical barriers and saving travel time (56). Currently, the number of psycho-oncological pilot studies of online interventions through videoconference in psycho-oncology is increasing. Results have highlighted participant engagement and effectiveness (57, 58). Moreover, a recent randomized control study (59) found similar effectiveness of both face-to-face and online psychotherapy for patients with a diagnosis of cancer with cancer distress. Finally, we must emphasize that we are going to carry out a follow-up at 6 months to confirm whether the results obtained after treatment are maintained, improve, or worsen in both treatment conditions.

However, our study could have some possible limitations. The first limitation we expect to find is the possible dropout rate in both treatment conditions. Treatment efficacy studies have found a dropout rate of 30% (9), which can interfere with the development of the study as well as the type of statistical analysis that we can perform. Another limitation is that the final sample may be composed mainly of older adult women, and because both treatment conditions will be carried out through video conference, this may make it difficult for them to adhere to the treatment conditions. Finally, the sample may be composed mainly of women with breast cancer, which will prevent the generalization of the results to men with other cancer diagnoses.

In sum, coping with cancer implies suffering, pain, hopelessness, low meaning in life, anxiety, and depression in many people (1–3). For this reason, it is necessary to develop effective psychotherapeutic treatments for people with cancer and survivors. Thus, it is necessary to verify the effectiveness of MCP by comparing it to well-established treatments. This will allow us to confirm whether MCP can become another well-established treatment for people with cancer.

## Ethics Statement

The study involves clinical experimentation with human participants. Researchers adhere to the Helsinki Convention and the Madrid Declaration of the World Psychiatric Association on clinical research. This project has been approved by the Ethics and Research Committee of the Valencian Institute of Oncology Foundation (CEIM: 2019-17). All participants/participants will be volunteers and give their informed consent to participate in the study.

## Author Contributions

JM drafted the manuscript with important contributions from PL and VG. JM, in collaboration with PL, VG, RB, SP, RR, and AG-C, designed the study and participated in each of its phases. JM translated and adapted the FC program. All authors participated in the review and revision of the manuscript and have approved the final manuscript to be published.

## Conflict of Interest

The authors declare that the research was conducted in the absence of any commercial or financial relationships that could be construed as a potential conflict of interest.

## Publisher's Note

All claims expressed in this article are solely those of the authors and do not necessarily represent those of their affiliated organizations, or those of the publisher, the editors and the reviewers. Any product that may be evaluated in this article, or claim that may be made by its manufacturer, is not guaranteed or endorsed by the publisher.

## Abbreviations

MCP: Meaning-Centered Psychotherapy
RCT: Randomized Controlled Trial
TAU: Treatment as Usual
MCGP: Meaning-Centered Grief Psychotherapy
CBT: Cognitive Behavioral Therapy
CONSORT, Consolidated Standards of Reporting Trials SPIRIT, Standard Protocol Items: Recommendations for Interventional Trials
MLQ: Meaning in Life Questionnaire
MEMS: Multidimensional Existential Meaning Scale
OASIS: Overall Anxiety Severity and Impairment Scale
ODSIS: Overall Depression Severity and Impairment Scale
HS: Hopelessness Scale
PANAS: The Positive and Negative Affect Schedule
QLI: Quality of life index-Spanish version
OTSM: Opinion and Expectations of Treatment Scale
MINI-MAC: Mini-Mental Adjustment to Cancer Scale
PTGI: Posttraumatic Growth Inventory
MANOVA: Multivariate analysis of variance
ANOVA: Analysis of variance.

## References

[1] Hinz A Krauss O Hauss JP Höckel M Kortmann RD Stolzenburg JU . Anxiety and depression in cancer patients compared with the general population. Eur J Cancer Care. (2010) 19:522–9. 10.1111/j.1365-2354.2009.01088.x 20030697

[2] Cleeland CS. Symptom burden: multiple symptoms and their impact as patient-reported outcomes. J Natl Cancer Inst Monogr. (2007) 37:16–21. 10.1093/jncimonographs/lgm005 17951226

[3] Henson LA Maddocks M Evans C Davidson M Hicks S Higginson IJ. Palliative care and the management of common distressing symptoms in advanced cancer: pain, breathlessness, nausea and vomiting, and fatigue. J Clin Oncol. (2020) 38:905. 10.1200/JCO.19.00470 32023162 PMC7082153

[4] Lethborg C Kissane DW Schofield P. Meaning and purpose (MaP) therapy I: therapeutic processes and themes in advanced cancer. Palliat Support Care. (2019) 17:13–20. 10.1017/S1478951518000871 30600795

[5] Marco JH Cañabate M Llorca G Pérez S. Meaning in life moderates hopelessness, suicide ideation, and borderline psychopathology in participants with eating disorders: a longitudinal study. Clin Psychol Psychot. (2020) 27:146–58. 10.1002/cpp.2414 31765024

[6] Marco JH Alonso S Baños R. Meaning-making as a mediator of anxiety and depression reduction during cognitive behavioral therapy intervention in participants with adjustment disorders. Clin Psychol Psychot. (2021) 28:325–33. 10.1002/cpp.2506 32881109

[7] Breitbart W Rosenfeld B Pessin H Kaim M Funesti-Esch J Galietta M . Depression, hopelessness, and desire for hastened death in terminally ill patients with cancer. JAMA. (2000) 284:2907–11. 10.1001/jama.284.22.2907 11147988

[8] Breitbart W Rosenfeld B Gibson C Pessin H Poppito S Nelson C . Meaning-centered group psychotherapy for patients with advanced cancer: a pilot randomized controlled trial. Psycho-oncology. (2010) 19:21–8. 10.1002/pon.1556 19274623 PMC3648880

[9] van der Spek N Vos J van Uden-Kraan CF Breitbart W Cuijpers P Holtmaat K . Efficacy of meaning-centered group psychotherapy for cancer survivors: a randomized controlled trial. Psychol Med. (2017) 47:1990–2001. 10.1017/S0033291717000447 28374663 PMC5501751

[10] Frankl VE. Man's Search for Meaning. New York, NY: Simon and Shuster (1985).

[11] Frankl VE. The Doctor and the Soul. New York, NY: Knopf (1969/1986).

[12] Vos J Vitali D. The effects of psychological meaning-centered therapies on quality of life and psychological stress: a metaanalysis. Palliat Support Care. (2018) 16:608–32 10.1017/S1478951517000931 30246682

[13] Breitbart W Poppito S Rosenfeld B Vickers AJ Li Y Abbey J . Pilot randomized controlled trial of individual meaning-centered psychotherapy for patients with advanced cancer. J Clin Oncol. (2012) 30:1304–9. 10.1200/JCO.2011.36.2517 22370330 PMC3646315

[14] Rosenfeld B Saracino R Tobias K Masterson M Pessin H Applebaum A . Adapting meaning-centered psychotherapy for the palliative care setting: results of a pilot study. Palliat Med. (2017) 31:140–6. 10.1177/0269216316651570 27435603 PMC5473503

[15] Lichtenthal WG Catarozoli C Masterson M Slivjak E Schofield E Roberts KE . An open trial of meaning-centered grief therapy: rationale and preliminary evaluation. Palliat Support Care. (2019) 17:2–12. 10.1017/S1478951518000925 30683164 PMC6401220

[16] Applebaum AJ Buda KL Schofield E Farberov M Teitelbaum ND Evans K . Exploring the cancer caregiver's journey through web-based meaning-centered psychotherapy. Psycho-Oncology. (2018) 27:847–56. 10.1002/pon.4583 29136682 PMC8045418

[17] Kearney JA Ford JS. Adapting Meaning-Centered Psychotherapy for Adolescents and Young Adults With Cancer: Issues of Meaning and Identity. In: Breitbart W editor. Meaning-centered psychotherapy in the cancer setting: Finding meaning and hope in the face of suffering. Oxford: England, Oxford University Press (2017). p. 100–11.

[18] Winger JG Ramos K Kelleher SA Somers TJ Steinhauser KE Porter LS . Meaning-centered pain coping skills training: a pilot feasibility trial of a psychosocial pain management intervention for patients with advanced cancer. J Palliat Med. (2022) 25:60–9. 10.1089/jpm.2021.0081 34388037 PMC8721493

[19] Torres-Blasco N Castro-Figuero E Garduño-Ortega O Costas-Muñiz R. Cultural adaptation and open pilot of meaning-centered psychotherapy for puerto rican patients with advanced cancer. Science. (2020) 8:100–7. 10.11648/j.sjedu.20200804.12 34532506 PMC8442672

[20] Steger F Frazier PA Zacchanini JL. Terrorism in two cultures: stress and growth following september 11 and the madrid train bombings. J Loss Trauma. (2021) 13:6511–27. 10.1080/15325020802173660

[21] Greer S Moorey S Baruch J. Evaluation of adjuvant psychological therapy for clinically referred cancer patients. Br J Cancer. (1991) 63:257–260. 10.1038/bjc.1991.60 1997103 PMC1971799

[22] Moorey S Greer S Bliss J Law M A A comparison of adjuvant psychological therapy and supportive counselling in patients with cancer. Psychooncology. (1998) 7:218–28. 10.1002/(SICI)1099-1611(199805/06)7:3&lt;218::AID-PON308&gt;3.0.CO;2-D 9638783

[23] de la Torre-Luque A Gambara H López E Cruzado JA. Psychological treatments to improve quality of life in cancer contexts: a meta-analysis. Int J Clin Health Psyc. (2016) 16:211–9. 10.1016/j.ijchp.2015.07.005 30487864 PMC6225027

[24] Barani M Bakhtiari M Firoozabadi VS Mehdizadeh M Sadeghi A. An evaluation of adjuvant psychological therapy (APT) effectiveness on the quality of life of patients with hematologic malignancies. Curr Psychol. (2019) 38:1728–35. 10.1007/s12144-017-9716-3

[25] Moher D Schulz KF Altman DG. The CONSORT statement: revised recommendations for improving the quality of reports of parallel group randomized trials. J Am Pediatr Med Assoc. (2001) 91:437–42. 10.7547/87507315-91-8-437 11574648

[26] Moher D Hopewell S Schulz KF Montori V Gotzsche PC Devereaux PJ . CONSORT 2010 explanation and elaboration: updated guidelines for reporting parallel group randomised trials. J Clin Epidemiol. (2010) 63:e1–37. 10.1016/j.jclinepi.2010.03.004 20346624

[27] Chan AW Tetzlaff JM Altman DG Laupacis A Gotzsche PC Krleza-Jeric K. SPIRIT 2013 statement: defining standard protocol ítems for clinical trials. Ann Intern Med. (2013) 158:200. 10.7326/0003-4819-158-3-201302050-00583 23295957 PMC5114123

[28] Chan AW Tetzlaff JM Gotzsche PC Altman DG Mann H Berlin JA . SPIRIT 2013 explanation and elaboration: guidance for protocols of clinical trials. BMJ Open. (2013) 346: e7586. 10.1136/bmj.e7586 23303884 PMC3541470

[29] Dietrich N Estrade A Cruzado AJ. Eficacia de la Psicoterapia Centrada en el Sentido en pacientes adultos con cáncer avanzado: revisión sistemática y meta-análisis. Psicooncologí*a*. (2021) 18:227–44. 10.5209/psic.77752

[30] Faul F Erdfelder E Lang A Buchner A. G^*^power 3: a flexible statistical power analysis program for the social, behavioral, and biomedical sciences. Behav Res Methods. (2007) 39:175–91. 10.3758/BF03193146 17695343

[31] Crumbaugh JC Maholick LT. Manual of Instructions for the Purpose in Life Test Saratoga. Viktor Frankl Institute of Logotherapy (1969).

[32] Breitbart WS Poppito SR. Meaning-Centered Group Psychotherapy for Patients With Advanced Cancer: A Treatment Manual. Oxford: Oxford University Press (2014).

[33] Bernal G. Intervention development and cultural adaptation research with diverse families. Fam Process. (2006) 45:143–51. 10.1111/j.1545-5300.2006.00087.x 16768015 PMC1693965

[34] Steger MF Frazier P Oishi S Kaler M. The meaning in life questionnaire: assessing the presence of and search for meaning in life. J Couns Psychol. (2006) 53:80–93. 10.1037/0022-0167.53.1.80

[35] George LS Park CL. The multidimensional existential meaning scale: a tripartite approach to measuring meaning in life. J Posit Psychol. (2017) 12:613–27. 10.1080/17439760.2016.1209546

[36] Norman SB Hami S Means-Christensen AJ Stein MB. Development and validation of an overall anxiety severity and impairment scale (OASIS). Depress Anxiety. (2006) 23:245–49. 10.1002/da.20182 16688739

[37] González-Robles A Mira A Miguel C Molinari G Díaz-García A García-Palacios A . Brief online transdiagnostic measure: psychometric properties of the overall anxiety severity and impairment scale (OASIS) among Spanish patients with emotional disorders. PLoS ONE. (2018) 13:e0206516. 10.1371/journal.pone.0206516 30383797 PMC6211825

[38] Ito M Bentley KH Oe Y Nakajima S Fujisato H Kato N . Assessing depression related severity and functional impairment: the overall depression severity and impairment scale (ODSIS). PLoS ONE. (2015) 10:1–14. 10.1371/journal.pone.0122969 25874558 PMC4395441

[39] Mira A González-Robles A Molinari G Miguel C Díaz-García A Bretón-López J . Capturing the severity and impairment associated with depression: the overall depression severity and impairment scale (ODSIS) validation in a Spanish clinical sample. Front Psychiatr. (2019) 10:180. 10.3389/fpsyt.2019.00180 31024352 PMC6465570

[40] Beck AT Weissman A Lester D Trexler L. The measurement of pessimism: the hopelessness scale. J Consult Clin Psych. (1974) 42:861–5. 10.1037/h0037562 4436473

[41] Viñas F Villar E Caparrós B Juan J Cornellá M Pérez I. Feelings of hopelessness in a Spanish university population: descriptive analysis and its relationship to adapting university, depressive symptomatology and suicidal ideation. Soc Psych Epid. (2004) 39:326–34. 10.1007/s00127-004-0756-2 15085336

[42] Watson D Clark LA Tellegen A. Development and validation of brief measures of positive and negative affect: The PANAS scales. J Pers Soc Psychol. (1988) 54:1063–70. 10.1037/0022-3514.54.6.1063 3397865

[43] Sandín B Chorot P Lostao L Joiner TE Santed ME Valiente R. Escalas PANAS de afecto positivo y negativo: validación factorial y convergencia transcultural. Psicothema. (1999) 11:37–51.

[44] Mezzich JE Cohen NL Ruiperez MA Banzato CE Zapata-Vega MI. The multicultural quality of life index: presentation and validation. J Eval Clin Pract. (2011) 17:357–64. 10.1111/j.1365-2753.2010.01609.x 21208350

[45] Mezzich JE Ruipérez MA Pérez C Yoon G Liu J Mahmud S. The Spanish version of the quality of life index: presentation and validation. J Nerv Ment Dis. (2000) 188:301–5. 10.1097/00005053-200005000-00008 10830568

[46] Borkovec TD Nau SD. Credibility of analogue therapy rationales. J Behav Ther Exp Psychiatr. (1972) 3:257–60. 10.1016/0005-7916(72)90045-6

[47] Watson M Law M Dossantos M Greer S Baruch J Bliss J. The mini-MAC: further development of the mental adjustment to cancer scale. J Psychosoc Oncol. (1994) 12:33–46. 10.1300/J077V12N03_03

[48] Andreu Y Murgui S Martinez P Romero R. Mini-mental adjustment to cancer scale: construct validation in Spanish breast cancer patients. J Psychosom Res. (2018) 114:38–44. 10.1016/j.jpsychores.2018.09.004 30314577

[49] Calhoun LG Tedeschi RG. The posttraumatic growth inventory: measuring the positive legacy of trauma. J Trauma Stress. (1996) 9:455–71. 10.1002/jts.2490090305 8827649

[50] Pajón L Greco AM Pereda N Gallardo-Pujol D. Factor structure of the Posttraumatic Growth Inventory in a Spanish sample of adult victims of interpersonal violence in childhood. J Psychopathol Clin Psychol. (2020) 25:101–110. 10.5944/rppc.26017

[51] Gueorguieva R Krystal JH. Move over ANOVA: progress in analyzing repeated-measures data and its reflection in papers published in the archives of general psychiatry. Arch Gen Psychiatry. (2004) 61:310–7. 10.1001/archpsyc.61.3.310 14993119

[52] Cohen J. Statistical Power Analysis for the Behavioral Sciences. 2nd ed. Hillsdale, NJ: Lawrence Erlbaum Associates Publishers (1988).

[53] Cooke A Smith D Booth A. Beyond PICO: the SPIDER tool for qualitative evidence synthesis. Qual Health Res. (2012) 22:1435–43. 10.1177/1049732312452938 22829486

[54] Levitt HM Motulsky S Wertz F Morrow S. Recommendations for designing and reviewing qualitative research in psychology: promoting methodological integrity. Qual Psychol. (2017) 4:2–22. 10.1037/qup0000082

[55] Lleras de Frutos M Casellas-Grau A Sumalla EC Gracia M Borràs JM Ochoa Arnedo C. A systematic and comprehensive review of internet use in cancer patients: psychological factors. Psychooncology. (2020) 29:6–16. 10.1002/pon.5194 31385400

[56] Gorlick A Bantum EOC Owen JE. Internet-based interventions for cancer-related distress: exploring the experiences of those whose needs are not met. Psychooncology. (2014) 23:452–8. 10.1002/pon.3443 24243756 PMC4167707

[57] Sansom-Daly UM Wakefield CE Bryant RA Patterson P Anazodo A Butow P . Feasibility, acceptability, and safety of the recapture life videoconferencing intervention for adolescent and young adult cancer survivors. Psychooncology. (2019) 28:284–92. 10.1002/pon.4938 30414219

[58] Zernicke KA Campbell TS Speca M McCabe-Ruff K Flowers S Carlson LE . Randomized wait-list controlled trial of feasibility and efficacy of an online mindfulness-based cancer recovery program: the eTherapy for cancer applying mindfulness trial. Psychosom Med. (2014) 76:257–67. 10.1097/PSY.0000000000000053 24804884

[59] Lleras de Frutos M Medina JC Vives J Casellas-Grau A Marzo JL Borràs JM Ochoa-Arnedo C. Video conference vs face-to-face group psychotherapy for distressed cancer survivors: a randomized controlled trial. Psychooncology. (2020) 29:1995–2003. 10.1002/pon.5457 32618395

